# Analysis of influence of physical health factors on subjective wellbeing of middle-aged and elderly women in China

**DOI:** 10.1186/s12889-022-12655-6

**Published:** 2022-06-06

**Authors:** Dong Wang, Hongxia Gao, Xin Xu, Dan Han, Kuan Yi, Guilin Hou

**Affiliations:** 1 School of Medicine and Health Management, Tongji Medical College, Huazhong University of Science and Technology, Wuhan, Hubei China; 2 Research Center for Rural Health Service, Key Research Institute of Humanities & Social Sciences of Hubei Province, Wuhan, Hubei China

## Abstract

**Purpose:**

Despite a maturing literature on the association between subjective wellbeing (SWB) and mental condition, little is known regarding the happiness–physical health relation in China, among middle-aged and elderly women (MAEW) in particular. This study aimed to understand the effect of physical health on the SWB of MAEW in China.

**Methods:**

Data from the 2014 and 2018 China Family Panel Study were used to analyse the SWB of women over the age of 45 years. In addition, descriptive statistics was used to describe the population distribution and panel ordered logit regression for regression analysis.

**Results:**

Majority of the respondents reported satisfactory SWB, and the proportion of the respondents who were very happy and happy was more than 68%. In terms of health factors, self-rated health, 2-week morbidity and BMI were significantly related to the SWB of MAEW (all *P*-values < 0.05). Physical exercise (*P*-value < 0.01) was positively associated with SWB, whereas smoking status and drinking status were not related to SWB. In addition, demographic indicators, such as registered residence (*P*-value < 0.01), income (*P*-value < 0.01) and social status (*P*-value < 0.01), significantly affected the SWB of MAEW.

**Conclusion:**

This study showed that MAEW’s physical health could affect their SWB. Increased attention should be paid to the physical health of MAEW to improve their SWB. Policy mechanisms could be designed to motivate MAEW to take the initiative to engage in regular physical activity to improve their SWB. In addition, increased attention be paid to groups with low socioeconomic status and high stress, especially those who are employed, to improve residents’ happiness.

**Supplementary Information:**

The online version contains supplementary material available at 10.1186/s12889-022-12655-6.

## Introduction

Subjective wellbeing (SWB) refers to a person’s self-evaluation of life quality, which is a comprehensive evaluation of emotions, including life satisfaction, happiness, pessimism and optimism [1]. This is a positive psychological state characterised by high level of life satisfaction, high level of positive emotion and low level of negative emotion. Philosophers have debated the nature of a good life for millennia and one conclusion that has emerged from this debate is that a good life is a happy life [2]. The United Nations considers SWB as an important component of life quality in its Human Development Report and suggested that member states include ‘happiness’ in their assessment of their ‘national development index’ [3]. As an indicator of the quality of life and psychological status of residents [4], SWB has also been incorporated into government policies globally and become an important performance indicator of general concern, such as in France [5] and Britain [6].

However, according to the World Happiness Report [7, 8], the happiness ranking of Mainland Chinese is consistently in the middle and lower ranges in the world. From 2013 to 2020, during the golden age of China’s rapid economic growth, the ranking of Mainland Chinese not only did not increase but even declined. Therefore, in China’s unique social environment, the status quo of SWB of some special Groups in China must be given attention. Middle-aged and elderly women (MAEW, aged 45 years and above) in China are a group worthy of study because they are experiencing a different social, work, family and living environment from other groups in China and similar groups in other countries.

Unlike other countries, Chinese women are expected by society to not only be responsible for their families but also perform well in the workplace [9]. They have a higher level of labour force participation than women in other countries. According to the International Labour Organisation, the labour participation rate of Chinese women was approximately 60% in 2020 [10]. However, influenced by traditional Chinese culture and Confucianism, Chinese women’s family responsibilities, such as cooking, doing laundry and caring for their children, persist [9, 11]. As a result, the MAEW in China are experiencing conflict between work and family [12]. If a woman marries at the age of 25, by the time she is 45, she has experienced the dual pressure of work and life for 20 years, and this pressure continues to increase over time. In terms of work, due to the potential conflict between the time devoted to taking care of family and work input, MAEW face greater competitive pressure and even the risk of dismissal and their work pressure is much higher than that of man and young women [13]. As for their family, aging parents and children’s heavy study pressure put forward high requirements for family care from women [14]. With regard to physiology, middle-aged women may be in menopause and their mental and physical conditions may be relatively fragile [15]. In addition to urban middle-aged women, rural middle-aged women do not have professional jobs and their participation in unstable agricultural labour could bring great pressure [16]. Moreover, rural areas are deeply influenced by traditional Chinese culture. Thus, the family status of middle-aged women in rural areas is lower than that of middle-aged women in cities.

The above evidence showed that Chinese MAEW have been under great pressure and time sacrifice for a long time. Under this heavy pressure, MAEW may not have sufficient time for sleep and physical exercise and their lifestyles [17] and health behaviour may change, which could lead to a decline in health and an increase in chronic diseases [18, 19]. Due to the irreversible characteristics of health damage, this health status of MAEW could continue to affect the whole stage of old age. According to a survey of middle-aged and older adults in China, depression and obesity in middle-aged and elderly Chinese women are significantly higher than those in men of the same age group [20]. Furthermore, more than 75.8% of women over the age of 60 years have at least one chronic disease [21]. In China’s social environment, studying the SWB level of MAEW and exploring the effect of health status and health behaviour on SWB are of great significance.

The past years has witnessed an emerging volume of literature exploring SWB. Most were limited to the relationship between SWB and personality [22], mental condition [23], subjective health [24] and socioeconomic status [25]. Research has found that positive SWB is beneficial for a number of life activities [26, 27] and it has a promoting effect on individual health and longevity [28–30]. However, research about the effects of physical health on happiness is limited. Therefore, the present study aimed to determine whether this group’s physical health status affects its SWB. Data from the China Family Panel Study (CFPS) were used to investigate the effect of physical health on the SWB of MAEW.

## Methods

### Data sources

The CFPS is a nationwide biennial household survey organised by the Institute of Social Science Survey of Peking University in 2010. The CFPS sample covers 25 provinces/municipalities/autonomous regions in China, with a target sample size of 16,000 households. The population in the survey area accounts for 94.5% of the total Mainland Chinese population and thus is highly representative. The Peking University Biomedical Ethics Review Committee provided ethical approval of the survey (approval number: IRB00001052-14,010). Respondents were given a statement explaining the purpose of the study and all study participants signed a written informed consent prior to being investigated.

Panel data from two waves of CFPS surveys were used (i.e. 2014 and 2018), with total sample sizes of 35,720, and 37,147, respectively. This study mainly examined the influence of physical health factors on the SWB of MAEW. Therefore, cases with missing values in physical health factors, SWB and demographic indicators were deleted. Ultimately, two phase-tracking samples of 4,997 individuals were obtained and a total of 9,994 observations were conducted.

### Variables and definitions

#### Dependent variable

The dependent variable was SWB. In the 2014 and 2018 CFPS surveys, the question to determine SWB is ‘How happy do you feel?’ In the 2014 and 2018 CFPS surveys, the answers range from 0 (lowest score) to 10 (highest score), with 0–6 denoting low SWB and 7–10 denoting high SWB, referring to the classification of the score (0–10) by the residents on the questionnaire [31, 32].

#### Physical health

The following five indicators were employed to evaluate physical health (health indicators that reflect body condition): self-reported health (SRH), 2-week morbidity (whether the participants felt physical discomfort in the past 2 weeks), BMI, chronic disease (whether the participants experienced a doctor-diagnosed chronic disease in the past 6 months) and hospitalisation (whether the participants were hospitalised due to illness in the past 12 months). In the CFPS questionnaire, the SRH was recorded on a five-point scale as follows: 1 = excellent, 2 = very good, 3 = good, 4 = not good and 5 = poor. Following Chinese standard practices, BMI was classified into four categories in accordance with the guidelines on the prevention and treatment of overweight and obesity amongst Chinese adults: BMI < 18.5 (underweight), 18.5 ≤ BMI < 24 (normal), 24 ≤ BMI < 28 (overweight) and BMI ≥ 28 (obese) [33].

#### Health behaviour

Three factors that may affect health were selected, namely, physical exercise, smoking status and drinking status. Physical exercise was measured by asking the participants the question ‘How often did you exercise in the past week?’ The responses were classified as ‘never’ (0 times) and ‘sometimes’ (≥ one time). Smoking status was measured by asking the participants whether they smoked in the past month (0 = ‘no’ and 1 = ‘yes’). Drinking status was measured by asking the participants whether they drank alcohol more than three times a week in the past month (0 = ‘no’ and 1 = ‘yes’).

#### Sociodemographic variables

Other factors that may affect individual SWB were selected, including age, marital status, educational level, income, social status, employment situation and registered residence. Age was classified into three categories as follows: 0 = 45–59 years, 1 = 60–74 years and 2 =  ≥ 75 years. Marital status was coded as 0 = never married, widowed or divorced or 1 = married. Education was classified into the following four levels: 0 = primary school and below, 1 = junior high school, 2 = senior high school and 3 = college or university and above. Employment situation was measured by asking the participants whether they were currently employed (0 = ‘no’ and 1 = ‘yes’). Registered residence was categorised as urban (1) or rural (0) and income and social status were measured by a score from 1 (lowest score) to 5 (highest score).

### Statistical analysis

As mentioned, the SWB of MAEW was measured from 0 to 10 as an ordinal categorical variable; thus, using the panel ordered logit regression for empirical analysis was appropriate. The SWB of MAEW was the dependent variable Y, which is an ordered variable with k levels (k = 11 in this paper). In addition, this study tested the robustness of the model by using the fixed effects model.

The panel ordered logit model was established on the basis of a potential variable $happiness$, which was replaced by $h$ below. The setting of the panel model for the latent variable $h$ is generally as follows:

1 $${h}_{it}={{\beta }^{T}health}_{it}+{\alpha }^{T}{ptiv}_{it}+{\epsilon }_{it},$$

where i (i = 1,2,3···) represents the individual, t is number of years, $\beta$ and $\alpha$ are parameters and ${\epsilon }_{it}$ is a random error term subject to logical distribution. In addition, ${health}_{it}$ and ${ptiv}_{it}$ are independent variables, where ${health}_{it}$ represents the health-related indicators for individual i at time t and ${ptiv}_{it}$ represents demographic indicators for individual i at time t.

The relationship between the latent variable ${h}_{it}$ and the ordered variable ${H}_{it}$ is as follows:

2 $${H}_{it}\left\{\begin{array}{c}0,{h}_{it}\le {c}_{0}\\ 1,{c}_{0}<{h}_{it}\le {c}_{1}\\ 2,{c}_{1}<{h}_{it}\le {c}_{2}\\ 3,{c}_{2}<{h}_{it}\le {c}_{3}\\ 4,{c}_{3}<{h}_{it}\le {c}_{4}\\ 5,{c}_{4}<{h}_{it}\le {c}_{5}\\ 6,{c}_{5}<{h}_{it}\le {c}_{6}\\ 7,{c}_{6}<{h}_{it}\le {c}_{7}\\ 8,{c}_{7}<{h}_{it}\le {c}_{8}\\ 9,{c}_{8}<{h}_{it}\le {c}_{9}\\ 10,{c}_{9}<{h}_{it}\le {c}_{10}\end{array}\right.$$

${c}_{j}$ is the threshold value, which is the value of the ordered variable ${H}_{it}$.

Further, we can transform this equation into the following equation:

3 $${H}_{it}=j, {c}_{j-1}<{h}_{it}\le {c}_{j}, j=\mathrm{1,2},\mathrm{3,4},\mathrm{5,6},\mathrm{7,8},\mathrm{9,10}$$

The panel ordered logit model is defined as:

4 $$p\left({H}_{it}=j\right)=p\left( {c}_{j-1}<{h}_{it}\le {c}_{j}\right)=p\left( {c}_{j-1}-{{\beta }^{T}health}_{it}-{\alpha }^{T}{ptiv}_{it}<{h}_{it}\le {c}_{j}-{{\beta }^{T}health}_{it}-{\alpha }^{T}{ptiv}_{it}\right)=F\left( {c}_{j}-{{\beta }^{T}health}_{it}-{\alpha }^{T}{ptiv}_{it}\right)-F({c}_{j-1}-{{\beta }^{T}health}_{it}-{\alpha }^{T}{ptiv}_{it})$$

where $F\left(\right)$ is the cumulative distribution function of the logistic distribution.

## Results

### General characteristics of participants

Table 1 shows the frequency and distribution of the characteristics of the participants of the 2014 and 2018 CFPS surveys. Majority of the respondents reported satisfactory SWB, with more than 65% of the respondents in the two surveys claiming to have high SWB. In terms of health factors, approximately 45% of the respondents believed their health was poor and more than 43% felt physical discomfort within 2 weeks. In addition, more than 60% of the respondents were overweight. In terms of health behavioural factors, the data in 2018 showed that more than half of the respondents performed physical exercise. More than 95% of the respondents said they did not smoke nor drink.

**Table 1:** Characteristics of the study sample

Characteristic	Group	2014		2018	
		Number/ Mean	%/S.D	Number/ Mean	%/S.D
Subjective Well-being	0–6	1555	31.1	1580	31.6
	7–10	3442	68.9	3417	68.4
Age	45–59	3196	64.0	2458	49.2
	60–74	1665	33.3	2230	44.6
	≥ 75	136	2.72	309	6.2
registered residence	urban	2327	46.6	2470	49.4
	rural	1670	53.4	2527	50.6
education status	primary school and below	2845	56.9	3289	65.8
	junior high school	1078	21.6	1086	21.7
	Senior high school	506	10.1	506	10.1
	college or university degree and above	568	11.4	116	2.4
marital status	Married	4439	88.8	4256	85.2
	unmarried	558	11.2	741	14.8
SRH	very good	415	8.3	474	9.5
	good	721	14.4	456	9.1
	acceptable	1707	34.2	1804	36.1
	bad	936	18.7	846	16.9
	very bad	1218	24.4	1417	28.6
chronic disease	Yes	1340	26.8	1448	29.0
	No	3657	73.2	3549	71.0
hospitalization	Yes	679	13.6	960	19.2
	No	4318	86.4	4037	80.8
two-week morbidity	Yes	2148	43.0	2219	44.4
	No	2849	57.0	2778	55.6
Physical exercise	Yes	1941	38.8	2636	52.7
	No	3056	61.2	2361	47.3
Drinking status	Yes	170	3.4	183	3.7
	No	4827	96.6	4814	96.3
Smoking status	Yes	232	4.6	225	4.5
	No	4765	95.4	4772	95.5
Working situation	Yes	3348	67.0	3035	60.7
	No	1649	33.0	1962	39.3
BMI	obesity	368	7.4	319	6.3
	overweight	2663	53.3	2495	50.0
	normal weight	1562	31.2	1704	34.1
	underweight	404	8.1	479	9.6
Income status		2.50	± 1.06	3.02	± 1.16
Social status		3.08	± 1.04	3.31	± 1.14

### Panel ordered logit regression analysis of SWB

Table 2 presents the results of the panel ordered logit regression analysis. SRH and BMI were positively related to the SWB of MAEW and 2-week morbidity was negatively correlated with SWB. Moreover, physical exercise was positively associated with SWB, whereas smoking status and drinking status had no statistically significant effect on the SWB of MAEW. In addition, in terms of individual characteristics, the higher the residents’ income and social status, the higher their likelihood of having high SWB. The respondents who were married, unemployed and living in an urban area had higher SWB than those who were unmarried, employed and living in a rural area; the difference was statistically significant. Finally, educational level had no statistically significant effect on the SWB of MAEW.

**Table 2:** Panel ordered Logit regression analysis of subjective well-being

variable	Coef	Std. Err	*P* value	[95% Conf. Interval]
Age				
45–59	0.748	0.130	< 0.001	0.493 ~ 1.003
60–74	0.212	0.053	< 0.001	0.108 ~ 0.317
≥ 75				
Registered residence				
urban	0.171	0.054	0.001	0.065 ~ 0.276
rural				
Education status				
college or university degree and above	-0.063	0.090	0.483	-0.240 ~ 0.113
Senior high school	-0.144	0.087	0.100	-0.317 ~ 0.027
junior high school	-0.017	0.065	0.783	-0.145 ~ 0.109
primary school and below				
Marital status				
Married	0.521	0.076	< 0.001	0.371 ~ 0.670
unmarried				
Working situation				
Yes	-0.314	0.054	< 0.001	-0.421 ~ -0.208
No				
SRH				
very bad	-1.155	0.098	< 0.001	-1.347 ~ -0.962
bad	-1.032	0.096	< 0.001	-1.221 ~ -0.843
acceptable	-0.835	0.088	< 0.001	-1.007 ~ -0.663
good	-0.391	0.100	< 0.001	-0.588 ~ -0.195
very good				
chronic disease				
Yes	-0.001	0.053	0.991	-0.104 ~ 0.103
No				
Hospitalization				
Yes	0.046	0.062	0.454	-0.075 ~ 0.169
No				
two-week morbidity				
Yes	-0.158	0.049	0.001	-0.255 ~ -0.062
No				
Physical exercise				
Yes	0.229	0.046	< 0.001	0.135 ~ 0.317
No				
Drinking status				
Yes	0.003	0.123	0.978	-0.238 ~ 0.245
No				
Smoking status				
Yes	0.224	0.119	0.060	-0.009 ~ 0.458
No				
BMI				
obesity	0.437	0.118	< 0.001	0.205 ~ 0.670
overweight	0.357	0.098	< 0.001	0.164 ~ 0.549
normal weight	0.256	0.092	0.006	0.074 ~ 0.438
underweight				
Income status	0.123	0.022	< 0.001	0.079 ~ 0.167
Social status	0.327	0.023	< 0.001	0.281 ~ 0.374
Cut1	-5.249	0.236		-5.711 ~ -4.785
Cut2	-4.216	0.196		-4.600 ~ -3.831
Cut3	-3.305	0.180		-3.658 ~ -2.951
Cut4	-2.506	0.173		-2.845 ~ -2.165
Cut5	-2.091	0.171		-2.426 ~ -1.756
Cut6	-0.252	0.167		-0.579 ~ 0.075
Cut7	0.254	0.167		-0.072 ~ 0.581
Cut8	0.735	0.167		0.407 ~ 1.062
Cut9	2.005	0.169		1.674 ~ 2.334
Cut10	2.506	0.169		2.174 ~ 2.838

Model Wald chi2 = 846.74; Log likelihood = -17,990.021; The number of ‘cut’ in the regression is related to the number of dependent variable classifications. As an auxiliary parameter, the parameter value of ‘cut’ could be interpreted as which value is needed to enter the corresponding dependent variable category. The SWB of MAEW was measured from 0 to 10 as an ordinal categorical variable. Therefore, this study had 10 auxiliary parameters (cut1–10)

## Discussion

This study used two wave data of CFPS (a nationally representative sample of older Chinese) to explore the causal effects of different intensities of physical exercise on successful ageing. The panel ordered logit regression was adopted for statistical analysis. This study also controlled for various confounding factors.

The results showed that majority of the respondents reported satisfactory SWB and those who had high SWB was more than 68%. This study also revealed that physical health was related to SWB.

Firstly, the MAEW with low self-rated health and those who felt unwell in the past 2 weeks had low SWB. This finding indicated that physical health could have certain effects on SWB [28]. However, the present study also found no significant correlation between chronic diseases, hospitalisation and SWB. Other studies have found similar results. Some scholars [34] also revealed that when controlling for different measures of subjective health status, the effect size between chronic disease and SWB decreased or was not even significant. This result may indicate that patients with long-term illness have adapted to the related effects of the disease. Thus, their SWB is not affected by chronic diseases. People with high BMI generally tended to have a low level of happiness [35, 36], which contradicted the results of the present study. The reason for this result may be because the subjects of the present study were MAEW in China. As Chinese traditional culture believes that being fat means being healthy, MAEW may believe that the fatter they are, the higher their quality of life is [31].

Secondly, weekly physical exercise had a positive effect on the SWB of MAEW. Studies have found that physical exercise could improve individual health, which in turn, could enhance SWB [37]. Evidence showed that most people with the initiative to exercise have an optimistic attitude and participating in physical exercise could enhance an individual’s sense of pleasure [38], thereby improving SWB. Gremeaux V et al. [39] found that moderate-intensity exercise not only could partially reverse the effect of the ageing process on the physiological function but also likely to yield emotional benefits. Unfortunately, heavy burden from family and work could limit the time and energy of MAEW in China for conducting physical exercise, which may lead to low health status, resulting in low level of SWB. Therefore, the government and health policymakers need to consider measures, such as designing some incentive systems and building some sports venues, to promote physical exercise participation amongst MAEW groups to improve the SWB of these groups.

Thirdly, the MAEW with high income and social status had high SWB, which was consistent with the findings of Anderson C et al. [40] and Duffy RM et al. [41]. However, many researchers believed that after monthly income reaches a certain high level, SWB may decline [42, 43]. This phenomenon was not observed in the present study, thereby indicating that the overall income of MAEW in China remains relatively low.

In addition, this study found that smoking and drinking had no statistically significant effect on the SWB of MAEW, different from the findings of some studies in other countries [44, 45]. The reason for this outcome may be because in the Confucian society of China, a certain level of discrimination and antipathy exists towards women smoking and drinking; thus, women’s drinking and smoking have been viewed as pathological and women’s pleasure from drinking and smoking has been overlooked.

## Limitations

Firstly, the variables were obtained from questionnaire surveys and the indicators were answered subjectively by the respondents. Thus, the data may be affected by the respondents’ recall bias and the bias of the interviewers. Secondly, on the basis of the existing health indicators in the CFPS surveys, the selection of physical health-related variables was limited, indicating that other physical health indicators could not be included. Thirdly, owing to data constraints, some important variables (e.g. environment, children’s situations and family property) that may affect the relationship between physical health and SWB were not included in the analysis. Therefore, in future studies, researchers could reduce the bias of respondents by adding objective indicators and improve the index system in the questionnaire to make the effect of physical health on SWB more scientific.

## Conclusions

This study uses data from a large nationwide panel survey (i.e., the CFPS) to analyze the impact of physical health on the SWB of MAEW. We find that physical factors have an impact on SWB, and demographic indicators such as registered residence, income, and social status also have a significant effect on the SWB of MAEW. These findings provide strong policy support for the government to improve the SWB of the public. Based on the results of our study, the government should pay increased attention to the SWB of MAEW, especially those who are currently employed, and promote the improvement of SWB by improving residents’ health, social, and economic status. Moreover, the government should build additional sports facilities and encourage residents to engage in physical exercise, which can improve their health and increase their happiness. In addition, to increase the credibility of the results of our study, we use a fixed effects model to test robustness, and the results are consistent with the results of the panel ordered logit regression. This outcome shows that the research results are reliable.

## Supplementary Information

**Additional file 1.** 

**Additional file 2: Table 1.** Fixed effects model analysis of subjective well-being.

## Data Availability

The datasets generated and/or analysed during the current study are available in the CFPS Public Data repository, http://isss.pku.edu.cn/cfps/download/index#/fileTreeList.

## Abbreviations

SWB: Subjective Wellbeing
MAEW: Middle-aged And Elderly Women
CFPS: China Family Panel Study
