# Supplementary material for: Analysis of influence of physical health factors on subjective wellbeing of middle-aged and elderly women in China
Source: BMC Public Health. 2022 Jun 6;22:1127. doi: 10.1186/s12889-022-12655-6 (PMC9169341; doi:10.1186/s12889-022-12655-6)
Supplement: Supplementary file 2 — Additional file 2: Table 1. Fixed effects model analysis of subjective well-being. [file 12889_2022_12655_MOESM2_ESM.docx]

Table 1 presents the results of the fixed effects model analysis. The results are consistent with the results of the panel ordered logit regression. This outcome shows that the research results are reliable.

**Table 1 Fixed effects model analysis of subjective well-being**

| variable | Coef. | Std. Err. | P value | [95% Conf. Interval] |
| --- | --- | --- | --- | --- |
| Age |  |  |  |  |
| 45-59 | 0.724 | 0.120 | <0.001 | 0.489～0.959 |
| 60-74 | 0.219 | 0.050 | <0.001 | 0.120～0.317 |
| ≥75 |  |  |  |  |
| Registered residence |  |  |  |  |
| urban | 0.190 | 0.051 | 0.001 | 0.089～0.289 |
| rural |  |  |  |  |
| Education status |  |  |  |  |
| college or university degree and above | -0.027 | 0.085 | 0.483 | -0.194～0.140 |
| Senior high school | -0.044 | 0.085 | 0.100 | -0.210～0.122 |
| junior high school | 0.038 | 0.062 | 0.783 | -0.083～0.159 |
| primary school and below |  |  |  |  |
| Marital status |  |  |  |  |
| Married | 0.522 | 0.072 | <0.001 | 0.381～0.663 |
| unmarried |  |  |  |  |
| Working situation |  |  |  |  |
| Yes | -0.290 | 0.051 | <0.001 | -0.390～-0.189 |
| No |  |  |  |  |
| SRH |  |  |  |  |
| very bad | -0.957 | 0.089 | <0.001 | -1.131～-0.782 |
| bad | -0.805 | 0.088 | <0.001 | -0.977～-0.632 |
| acceptable | -0.630 | 0.079 | <0.001 | -0.785～-0.474 |
| good | -0.224 | 0.091 | 0.014 | -0.402～-0.045 |
| very good |  |  |  |  |
| chronic disease |  |  |  |  |
| Yes | 0.009 | 0.051 | 0.866 | -0.092～0.109 |
| No |  |  |  |  |
| Hospitalization |  |  |  |  |
| Yes | 0.028 | 0.059 | 0.630 | -0.087～0.144 |
| No |  |  |  |  |
| two-week morbidity |  |  |  |  |
| Yes | -0.190 | 0.047 | 0.001 | -0.282～-0.098 |
| No |  |  |  |  |
| Physical exercise |  |  |  |  |
| Yes | 0.238 | 0.044 | <0.001 | 0.151～0.324 |
| No |  |  |  |  |
| Drinking status |  |  |  |  |
| Yes | -0.035 | 0.116 | 0.761 | -0.263～0.193 |
| No |  |  |  |  |
| Smoking status |  |  |  |  |
| Yes | 0.164 | 0.111 | 0.137 | -0.052～0.381 |
| No |  |  |  |  |
| BMI |  |  |  |  |
| obesity | 0.454 | 0.113 | <0.001 | 0.234～0.675 |
| overweight | 0.344 | 0.093 | <0.001 | 0.161～0.527 |
| normal weight | 0.266 | 0.089 | 0.003 | 0.092～0.439 |
| underweight |  |  |  |  |
| Income status | 0.117 | 0.021 | <0.001 | 0.076～0.157 |
| Social status | 0.277 | 0.022 | <0.001 | 0.235～0.320 |
